# Prevalence of Frailty in Latin America and the Caribbean: A Systematic Review and Meta-Analysis

**DOI:** 10.1371/journal.pone.0160019

**Published:** 2016-08-08

**Authors:** Fabiana Araújo Figueiredo Da Mata, Priscilla Perez da Silva Pereira, Keitty Regina Cordeiro de Andrade, Ana Claudia Morais Godoy Figueiredo, Marcus Tolentino Silva, Maurício Gomes Pereira

**Affiliations:** 1 Department of Medical Sciences, University of Brasilia, Brasilia, Federal District, Brazil; 2 Department of Health Sciences, University of Brasilia, Brasilia, Federal District, Brazil; 3 Department of Health Sciences, Federal University of Amazonas, Manaus, Amazonas, Brazil; University of Exeter, UNITED KINGDOM

## Abstract

**Background:**

Countries in Latin America and the Caribbean (LAC) have experienced a rapid increase in their proportion of older people. This region is marked by a high prevalence of chronic diseases and disabilities among aging adults. Frailty appears in the context of LAC negatively affecting quality of life among many older people.

**Aim:**

To investigate the prevalence of frailty among community-dwelling older people in LAC through a systematic review and meta-analysis.

**Methods:**

A literature search was performed in indexed databases and in the grey literature. Studies investigating the prevalence of frailty with representative samples of community-dwelling older people in Latin America and the Caribbean were retrieved. Independent investigators carried out the study selection process and the data extraction. A meta-analysis and meta-regression were performed using STATA 11 software. The systematic review was registered at the International Prospective Register of Systematic Reviews under the number CRD42014015203.

**Results:**

A total of 29 studies and 43,083 individuals were included in the systematic review. The prevalence of frailty was 19.6% (95% CI: 15.4–24.3%) in the investigated region, with a range of 7.7% to 42.6% in the studies reviewed. The year of data collection influenced the heterogeneity between the studies.

**Conclusion:**

Frailty is very common among older people in LAC. As a result, countries in the region need to adapt their health and social care systems to demands of an older population.

## Introduction

Frailty is characterized by an accelerated decrease in several inter-related physiological systems resulting in the malfunction of homeostatic mechanisms [1]. This condition is more prevalent among older people, negatively affects people’s quality of life, and predicts disability, falls, hospitalization, and mortality [2, 3]. As a result, frail older people require extra care, which impacts individual and governmental financial planning [4].

Frailty has been studied extensively in recent years, and its prevalence has been investigated more thoroughly in North America, Europe, and developed countries, where it has appeared to increase with age and be higher among women [4, 5]. However, there is no consensus regarding the prevalence of frailty worldwide [4].

The lack of agreement regarding the best frailty measurements and diagnostic criteria has also been stated in the literature [6, 7]. Some well accepted conceptual models define frailty as a purely physical syndrome, while others include psychological and social aspects in its definition [3, 6, 8]. Based on these conceptual models, a variety of instruments have been developed to assess frailty. The Frailty Phenotype, for instance, classified frailty based on five physical criteria, while the Tilburg Frailty Indicator and the Frailty Index added social and psychological domains to their definition of frailty [3, 8–9].

The Frailty Phenotype is the most commonly used way of measuring frailty. It was developed and operationalized by Fried et al. (2001) and used data from the Cardiovascular Health Study (CHS) Cohort [3]. However, modified versions of the proposed Phenotype have often been used because it is not always feasible to assess all the physical criteria in the same way measured in the CHS Cohort [10]. As different conceptual models influence the selected characteristics for defining frailty [7], it has been observed that the prevalence of frailty varies according to each adopted definition and way of measurement [4, 10–12].

Few studies investigating the prevalence of frailty in less-developed countries are found in the literature. Countries in Latin American and the Caribbean (LAC) are experiencing a rapid increase in the proportion of aging citizens and this process is likely to continue for the next three decades [13–14]. Rising longevity in countries with poor standards of living increases the likelihood of having a larger population of frail older adults [13–14]. Moreover, compared to developed nations, Latin American adults are facing a higher number of chronic diseases and disabilities as they age [12, 15–16]. A study conducted in low- and middle-income countries reported similar or higher incidence of dementia compared with countries of high-income [17]. As a result, Latin American countries will need to adapt their institutions and public policies to the new challenges that arise from a less healthy older population [5, 12, 15].

Stating the prevalence of frailty is challenging due to the variety of frailty measurements. However, in an under researched region where an aging population is combined with marked social disadvantages having an estimated prevalence may contribute to planning health and social care policies. Some studies have investigated frailty in LAC cities, but, as far we are aware, no systematic review on the topic has been carried out thus far. Therefore, this study aims to investigate the pooled prevalence of frailty among community-dwelling older people in LAC through a systematic review and meta-analysis.

## Materials and Methods

### Register and protocol

This study was registered at Prospero (International Prospective Register of Systematic Reviews) under the number CRD42014015203.

### Eligibility criteria

We selected cross-sectional surveys and baseline assessments of longitudinal studies with representative samples of community-dwelling older men and women aged 60 years and above living in the Latin American and Caribbean region. Eligible studies had to report their working definition of frailty, to state the prevalence of frailty or to supply data that allowed us to calculate frailty prevalence measures. We excluded studies stating mean frailty scores without cutoff points for frailty categories and studies that examined a disease-specific population. The definition of frailty and sample size were not part of the criteria for excluding studies in this review. There was no limit for language, publication date or status. The minimum age of 60 years in reference to the older population was assumed according to the cutoff agreed to by the United Nations [18].

### Information sources and search strategy

The literature search for potential eligible studies was performed between 5^th^ and 7^th^ May 2016 using the following electronic databases: MEDLINE (via PubMed), Embase, Lilacs, SciELO, Google Scholar, Web of Science, Scopus, ProQuest, CINAHL, and academic works (theses database). Moreover, studies were also selected through manual search of reference citations. The search strategy was developed using Mesh terms for PubMed, EMTREE terms for Embase, and a combination of keywords. For example, the full electronic search strategy used at PubMed was: (“Frail Elderly” [Mesh] OR "Frail Elderly” [TIAB] OR “Frailty” OR “Frail Older People”) AND ("Prevalence" OR “Frequency”). The search strategy was slightly modified based on each database (S1 Table).

### Study selection process

The study selection process was carried out in two stages by four independent investigators (FAFM, PPSP, KRCA, and ACGF), each record was independently read by two authors. Records (articles) were selected based on their titles and abstracts; duplicate records were excluded. The remaining records were read in their entireties, and those suitable for the review were selected. In cases where a consensus could not be reached by the two authors, a third author helped make a decision regarding the paper selection. Sometimes, a record could describe more than one study; thus, the total number of individual studies was considered at the end of the review. When different studies shared the same population, the study that provided the largest sample size or the one with more detailed information about the participants and frailty definition was chosen–these criteria have been used by other authors [4]. Studies using modified versions of the Frailty Phenotype (i.e., that adopted different metrics or criterion) were also selected.

### Data extraction and quality assessment of studies

Three authors (FAFM, PPSP, and KRCA) independently extracted data onto a standardized datasheet. In cases of disagreement, decisions were made by consensus. The data extracted included studies’ features, sample sizes, and prevalence of frailty. In cases that a record compared two prevalence measures from different definitions of frailty, the lower prevalence was the chosen one in order to be more conservative. We tried to contact all the correspondent authors to gather data to complete the data extraction form for each study.

The quality assessments of the studies were carried out based on a tool described by Munn et al., 2015 [19–20]. This tool includes nine items for critical appraisals of the methodological quality of studies reporting prevalence data. For each criterion met, the study received a “yes”. The total number of “yes” answers was counted per study. The larger the number of “yes”, the lesser the risk of bias in the study. As one of the items presented in the tool inquired about the validity of the methods used to identify the condition, we considered modified versions of the Frailty Phenotype as a valid method in this item.

### Data analysis

The main outcome in this review was the prevalence of frailty in older people in LAC with a 95% confidence interval (95% CI).

A meta-analysis of a random-effects model was chosen *a priori*. We used the *metaprop ftt* command in STATA (version 11.0) to perform the analysis as it incorporates the Freeman-Tukey double arcsine method to stabilize the variance [21–22]. The chi-squared test was applied to measure heterogeneity between studies at the p < 0.10 significance level. We adopted this p-value over the standard p < 0.05 to be more conservative as low power is attributed to the chi-squared test in meta-analyses when a small number of studies or studies of small sample size are considered [23]. The magnitude of inconsistency was measured using I-squared (I^2^) statistics. High heterogeneity was considered when I^2^ was over 75%, moderate when it was between 25% and 75%, and low when I^2^ was less than 25% [23–26].

To explore potential sources of heterogeneity, sensitivity and subgroup analyses of the prevalence were carried out. Subgroups were divided by sex (men versus women), region (North America versus Central America versus South America), frailty definition (Frailty Phenotype versus Modified Frailty Phenotype versus Edmonton Frailty Scale versus Five physical criteria), and country (Brazil versus other countries). A meta-regression was performed considering p < 0.05 to determine whether possible covariates such as the sample size, mean age, proportion of women, data collection year (represented by the last year of the data collection), and study quality could explain the heterogeneity between studies. Meta-regressions were carried out in each subgroup analysis as well [26]. Potential publication bias (or the small-studies effect) was analyzed using Funnel plots and the Egger’s test [26–28]. We used STATA software (version 11.0) for all the statistical analysis.

## Results

### Selection process and characteristics of studies

**Fig 1:**
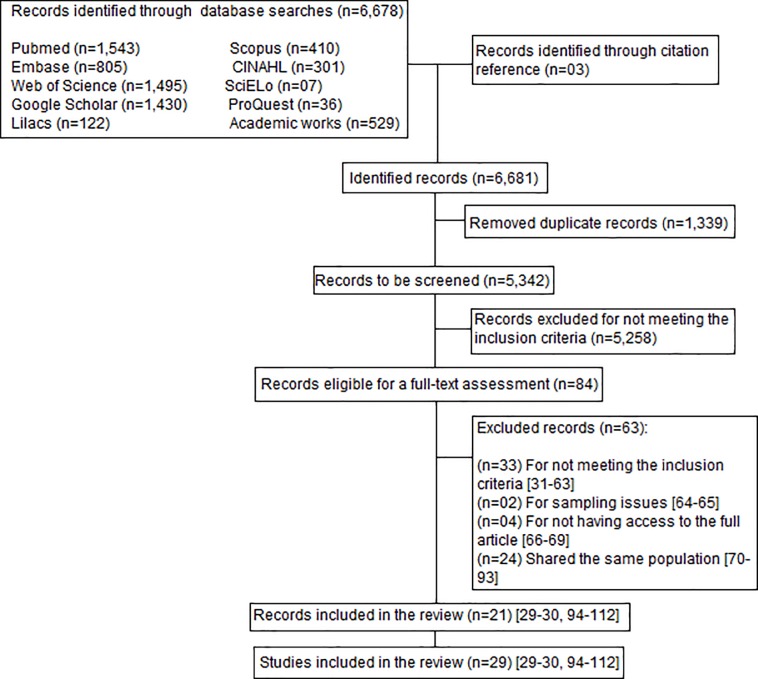
Flowchart of the study selection process.

The literature search yielded 6,678 records. After removing duplicates and assessing titles, abstracts, and inclusion criteria, 84 manuscripts were submitted for a complete reading. From these, 21 were included in the review. Two records [29–30] contained information on prevalence from four studies each. Therefore, a total of 29 studies were included in the systematic review. Fig 1 displays details about the selection process and the reasons for the exclusion of records [29–112].

Table 1 displays the characteristics of the studies [29–30, 94–112]. A total of 43,083 participants were included in the review; the majority of the studies were composed of women, and the feminine proportions in the samples ranged from 52.2% [97] to 67.7% [101]. Twenty two studies in this review used modified versions of the Frailty Phenotype to define frailty [29–30, 94–97, 99, 102–104, 108–109, 100–111], four studies used the Frailty Phenotype according to the operationalization used in the CHS [101, 106, 110, 112], two studies used the Edmonton Frailty Scale [98, 105], and one used five physical tests to define frailty [107]. Data were stratified by sex in 19 studies [29, 94–95, 97, 99, 101–104, 106–111]. Regarding geographic regions, 20 studies were performed in South America [29–30, 97, 101, 95–96, 98, 100, 102–106, 109–112]; four were performed in Central America [29–30, 107], and five were performed in North America [29–30, 94, 99, 108]. The quality assessment for each study is presented in the S2 Table. All studies seemed to be of good quality, with the number of “yes” answers varying between 7 and 9 with a mean of 8.3. A meta-analysis was performed for all of the 29 included studies. The data extraction form is presented in the S3 Table.

**Table 1:** Characteristics of the studies.

Author, publication year	Place	Year of data collection	Study group	Study design	Frailty definition	Sample size (n)	Mean age	Women (%)	Frailty Prevalence (%)	Confidence Interval (CI)
Aguilar-Navarro et al., 2015 [94]	Mexico	2001	Mexican Health and Aging Study (MHAS)	Baseline of a longitudinal study	Modified version of Frailty Phenotype	5,644	68.7	53.6	37.2	NA
Alvarado et al., 2008 [29]	Bridgetown, Barbados	1999–2000	SABE	Population-based	Modified version of Frailty Phenotype	1,446	NA	61.0	26.7	NA
Alvarado et al., 2008 [29]	São Paulo, Brazil	1999–2000	SABE	Population-based	Modified version of Frailty Phenotype	1,879	NA	59.0	40.6	NA
Alvarado et al., 2008 [29]	Santiago, Chile	1999–2000	SABE	Population-based	Modified version of Frailty Phenotype	1,220	NA	65.7	42.6	NA
Alvarado et al., 2008 [29]	Havana, Cuba	1999–2000	SABE	Population-based	Modified version of Frailty Phenotype	1,726	NA	62.8	39.0	NA
Alvarado et al., 2008 [29]	Mexico City, Mexico	1999–2000	SABE	Population-based	Modified version of Frailty Phenotype	1,063	NA	56.4	39.5	NA
Andrade et al., 2013 [95]	São Paulo, Brazil	2006	SABE—São Paulo	Cross-sectional	Modified version of Fried Phenotype	1,374	NA	59.7	8.5	NA
Corona et al., 2015 [96]	São Paulo, Brazil	2010	SABE—São Paulo	Cross-sectional	Modified version of Fried Phenotype	1,256	70.0	60.9	8.0	6.3–10.2
Curcio et al., 2014 [97]	Four cities in Colombia	2005	NA	Survey	Modified version of Frailty Phenotype	1,878	70.9	52.2	12.2	6.8–17.0
Fohn et al., 2013 [98]	Ribeirão Preto, Brazil	2010–2011	NA	Cross-sectional	Edmonton Frail Scale	240	73.5	62.9	39.2	NA
García-Peña et al., 2016 [99]	Mexico	2012	Mexican Health and Ageing Study (MHAS)	Cross-sectional	Modified version of Fried Phenotype	1,108	69.8	54.6	24.9	NA
Jotheeswaran et al., 2015 [30]	Cuba	2003–2007	10/66 Dementia Research Group’s	Population-based	Modified version of Frailty Phenotype	2,813	75.2	65.0	21.0	NA
Jotheeswaran et al., 2015 [30]	Domican Republic	2003–2007	10/66 Dementia Research Group’s	Population-based	Modified version of Frailty Phenotype	2,011	75.4	66.3	34.6	NA
Jotheeswaran et al., 2015 [30]	Venezuela	2003–2007	10/66 Dementia Research Group’s	Population-based	Modified version of Frailty Phenotype	1,997	72.3	63.2	11.0	NA
Jotheeswaran et al., 2015 [30]	Mexico	2003–2007	10/66 Dementia Research Group’s	Population-based	Modified version of Frailty Phenotype	2,003	74.2	Urban population: 66.5 Rural population: 60.9	Urban population: 10.1 Rural population: 8.5	NA
Jotheeswaran et al., 2015 [30]	Peru	2003–2007	10/66 Dementia Research Group’s	Population-based	Modified version of Frailty Phenotype	1,933	74.5	Urban population: 64.7 Rural population: 53.2	Urban population: 25.9 Rural population: 17.2	NA
Junior et al., 2914 [100]	Lafaiete Coutinho, Brazil	2011	Nutritional status, risk behaviors and health conditions of the elderly people of Lafaiete Coutinho-BA.	Cross-sectional	Modified version of Frailty Phenotype	286	NA	NA	23.8	NA
Neri et al., 2013 [101]	Belém, Brazil, Parnaíba, Brazil, Campina Grande, Brazil, Poços de Caldas, Brazil, Ermelino Matarazzo, Brazil, Campinas, Brazil, Ivoti, Brazil	2008–2009	FIBRA NETWORK	Cross-sectional	Fried Phenotype (CHS)	3,478	72.9	67.7	9.0	NA
Ocampo-Chaparro et al., 2013 [102]	Cali, Colombia	2009	NA	Population-based	Modified version of Frailty Phenotype	314	NA	NA	12.7	NA
Pegarori et al., 2014 [103]	Uberaba, Brazil	2012	FIBRA NETWORK	Cross-sectional	Modified version of Frailty Phenotype	958	73.8	64.4	12.8	10.87–15.11
Pinedo et al., 2010 [104]	Lima, Peru	NA	NA	Cross-sectional	Modified version of Frailty Phenotype	246	69.9	59.8	7.7	NA
Ramos et al., 2015 [105]	Montes Claros, Brazil	2013	NA	Population-based	Edmonton Frail Scale	639	70.6	64.0	33.6	NA
Ricci et al., 2014 [106]	Barueri, Brazil, Cuiabá, Brazil	2009–2010	FIBRA NETWORK	Population-based	Fried Phenotype (CHS)	761	71.9	64.3	9.7	NA
Rosero-Bixby et al., 2009 [107]	Costa Rica	2004–2006	CRELES	Baseline of a longitudinal study	Five physical tests: grip strength, pulmonary peak flow, standing up from a chair, picking an object up from the floor, and standing and walking 3m	2,827	NA	52.4	23.6	21.1–26.3
Ruiz-Arregui et al., 2013 [108]	Coyoacan, Mexico	2008–2009	Coyoacán Cohort Study	Baseline of a longitudinal study	Modified version of Frailty Phenotype	927	79.5	54.9	14.1	11.9–16.5
Samper-Ternent et al., 2016 [109]	Bogotá, Colombia	2012	SABE (Bogotá Study)	Cross-sectional	Modified version of Frailty Phenotype	1,442	70.7	61.0	9.4	NA
Sousa et al., 2012 [110]	Santa Cruz, Brazil	NA	FIBRA Network	Cross-sectional	Fried Phenotype (CHS)	391	74.0	61.4	17.1	NA
Tribess et al., 2012 [111]	Uberaba, Brazil	2010	Population Study of Physical Activityand Aging (*Estudo Populacional de Atividade Física e Envelhecimento*)	Cross-sectional	Modified version of Frailty Phenotype	622	71.0	65.0	19.9	NA
Vieira et al., 2013 [112]	Belo Horizonte, Brazil	2008–2009	FIBRA NETWORK	Population-based	Fried Phenotype (CHS)	601	74.3	66.2	8.7	NA

### Frailty Prevalence

**Fig 2:**
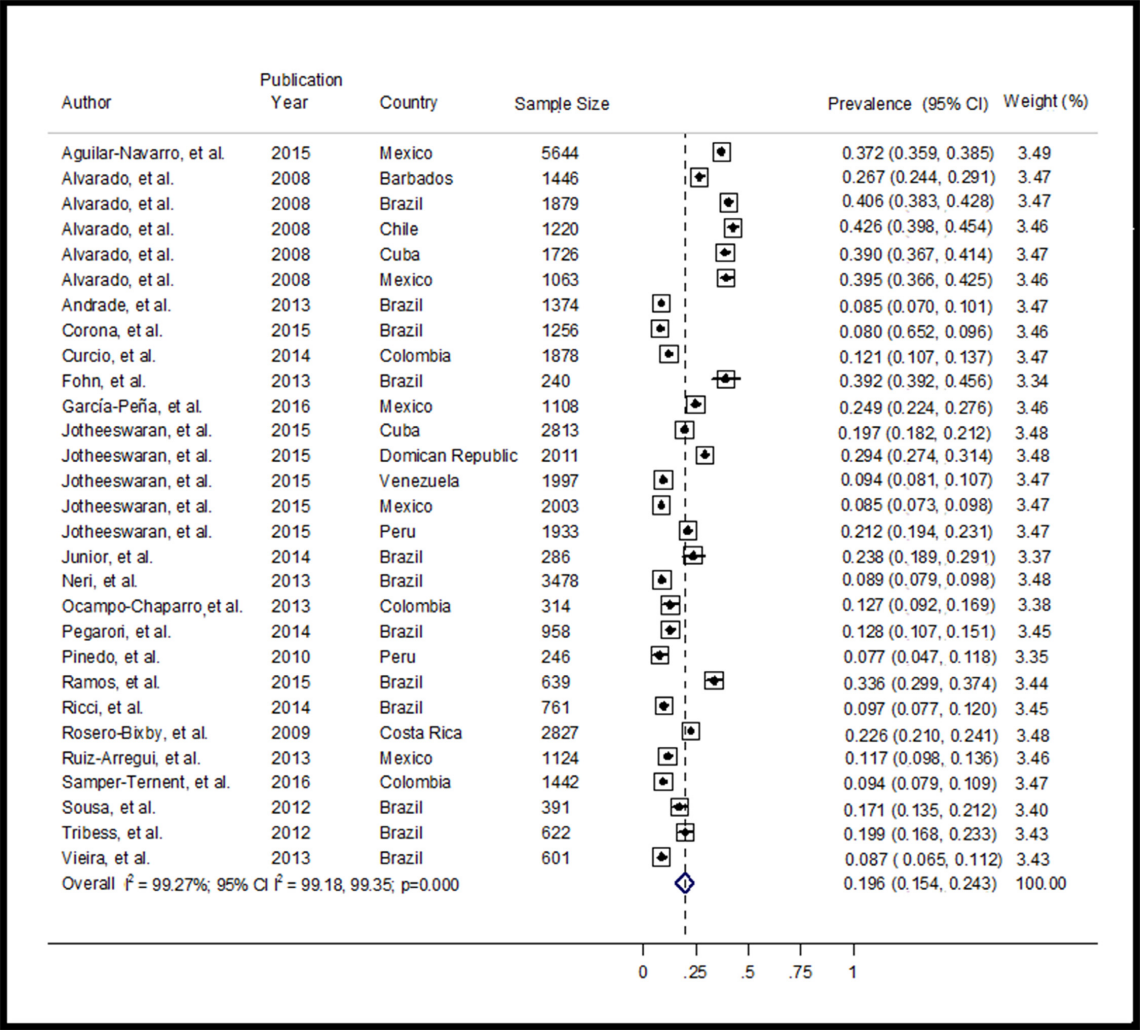
Meta-analysis of the prevalence of frailty in LAC.

The prevalence of frailty in Latin America and the Caribbean was 19.6% (95% CI: 15.4–24.3; 29 studies; 43,083 individuals; I^2^ = 99.3%, 95% CI: 99.18–99.35) with a range of 7.7% to 42.6% in the studies reviewed (Fig 2). Visual inspection of the funnel plot revealed asymmetry, and the Egger’s test findings suggested that publication bias might have been present (p = 0.040). We used the “trim and fill” approach to try to account for non-published results and the prevalence of frailty was 13.1% (95% CI = 8.2–17.9) [113]. Between-study heterogeneity was identified (Chi-square = 3848.02, df = 28, p<0.001). A meta-regression indicated that the year of data collection partly explained the heterogeneity observed in the prevalence of frailty (p = 0.003; R^2^ = 28.7%). S1, S2, and S3 Figs display the funnel plot, the trim and fill, and meta-regression graphs respectively.

### Sensitivity and subgroup analyses

We identified four studies with sample size larger than 2,500 which we considered outliers compared to the sample size of other studies included in this review [30, 94, 101, 107]. We performed a meta-analysis without these studies and the results were similar (Prevalence = 19.4%, CI = 14.8–24.5; I^2^ = 99.1, p<0.001).

A subgroup analysis revealed high heterogeneity in all analyzed categories, except for the one defining frailty according to the Edmonton Frailty Scale that presented moderate heterogeneity (Table 2). By considering the overlap among the confidence intervals in each subgroup, no differences in prevalence were observed for the population sex and for the country subgroup. However, frailty prevalence was higher in Central than South America, when the Edmonton Scale was used, and when modified versions of Frailty Phenotype were used compared to the traditional version.

**Table 2:** Subgroup analyses by sex, region, frailty definition, and country.

Subgroups	Number of studies [references]	Total number of participants	Frailty prevalence, % (95% CI)	I^2^(%)	p-value (chi-square)
**Sex**					
Female	19 [29, 94–95, 97, 99, 101–104,106–111]	17,669	23.4 (16.6–30.9)	99.2	<0.001
Male	19 [29, 94–95, 97, 99, 101–104, 106–111]	12,282	15.0 (11.1–19.4)	97.5	<0.001
**Region**					
North America	5[29–30, 94, 99, 108]	10,942	23.0 (10.9–38.0)	99.6	<0.001
Central America	4 [29–30, 107]	8,010	29.3 (22.6–36.4)	97.9	<0.001
South America	20 [29–30, 95–98, 100–106, 109–112]	21,515	17.1 (12.6–21.1)	99.0	<0.001
**Frailty definition**					
Frailty Phenotype	4 [101, 106, 110, 112]	5,231	10.6 (8.0–13.6)	86.8	<0.001
Modiefied Frailty Phenotype	22 [29–30, 94–97, 99–100, 102–104, 108–109, 111]	34,343	20.0 (15.0–25.5)	99.3	<0.001
Edmonton Frailty Scale	2 [98, 105]	879	35.8 (30.6–41.2)	56.9	0.128
Five physical tests	1 [107]	2,827	22.6 (21.1–24.2)	-	-
**Country**					
Brazil	12[29, 95–96, 98, 100–101, 103, 105–106, 110–112]	12,485	17.9 (11.3–25.6)	99.1	<0.001
Other countries	17[29–30, 94, 97, 99, 102, 104, 107–109]	30,795	20.9 (15.6–26.8)	99.3	<0.001

Meta-regressions performed in subgroups indicated that not all of the analyzed covariates were significantly possible causes of the high heterogeneity between the studies (p>0.05). However, the year of data collection partly explained the heterogeneity observed in the subgroups of women (p<0.001; R^2^ = 62.4%), men (p<0.05; R^2^ = 43.2%), in the modified version of Frailty Phenotype (p<0.001; R^2^ = 51.5%), and in the other countries subgroup (p = 0.001; R^2^ = 53.1%). Sample size, mean age, study quality, and the proportion of women were not found to explain the between-sample heterogeneity in any subgroup.

## Discussion

In LAC, on average, 19.6% of community-dwelling older people are frail. This prevalence ranges from 7.7% [104] to 42.6% [29] according to the studies selected for this review.

### Previous studies

The estimated prevalence of frailty in LAC is different to those observed in studies conducted in more developed regions. In 2012, a systematic review carried out with people aged 65 years and over in countries in Europe, North America, and Oceania investigated the average prevalence of frailty when using physical and broader definitions of the syndrome. The prevalence of physical frailty was 9.9%, and when broader definitions including psychological and social aspects were considered, the prevalence rose to 13.6% [4]. A cross-sectional analysis performed in 10 European countries revealed that 17% of the individuals aged at least 65 years were frail [5]. In a cohort study of community-dwelling older Japanese aged 65 years and above, the estimated prevalence of frailty was 12.5% [114].

A systematic review conducted with 21 studies from developing countries showed that measures of prevalence varied between 5.4% and 44.0% in community-dwelling older people aged 55 years and over. A summary measure of the prevalence was not estimated by the authors [115]. Another study carried out with people aged 50 years and over showed that lower income countries such as China, Ghana, India, Mexico, Russia Federation, and South Africa had lower levels of frailty compared to higher income countries from Europe [116].

We note that studies assessing frailty use different age cutoffs to classify older people. According to the World Health Organization, a minimum age of 65 years characterizes an older person in developed countries, while in developing countries this cutoff is 60 years and over [18]. Therefore, it is not possible to establish a direct comparison between the above referred prevalence measures and the one estimated in this review. However, in general, one may note a trend of lower prevalence of frailty in developed countries related to the estimated prevalence for LAC in this study.

Differences among frailty prevalence estimates between LAC and developed countries may be due to a number of factors. For instance, lifestyles, health statuses, and demographic and socioeconomic characteristics vary greatly between countries at different stages of development. In LAC, approximately 1 out of 5 older persons is frail. This large proportion of frail older people is likely to increase the demand for health and social services.

Our results showed no differences in prevalence between men and women. However, the literature shows that women are commonly more frail than men [5, 29, 117–120]. Because women live longer and generally have a larger number of comorbidities [121], a greater prevalence of frailty is expected in the female population. A study conducted in Europe showed that while women have a shorter disability-free life expectancy, men have a shorter life expectancy with frailty [122]. We could not assess the prevalence of frailty by age group in this review because the studies report different age categories and distributions. However, it is well established in the literature that frailty increases with age [29, 119–120] because as people age, they accumulate deficits and become more vulnerable to adverse health outcomes [7].

The prevalence of frailty in Central America was higher than in South America. However, this result should be interpreted with caution given the greater number of studies conducted in South America compared with the smaller number of studies conducted in Central America. The variability of sample size in the studies might have also contributed to an unbalanced comparison between the regions. The 19.6% estimated prevalence of frailty in LAC ranges from 7.7% to 42.6% among the individual studies selected in the review. These prevalence measures are from an array of studies conducted in different cities in LAC and show how varied the prevalence of frailty can be among individual studies in the region.

High levels of heterogeneity were observed between almost all of the prevalence measures in this review. The exception was the subgroup that defined frailty using the Edmonton Frailty Scale that presented non-significant heterogeneity. However, this result should not be automatically interpreted as between-study homogeneity because of the small number of studies in the subgroup–only two [23]. Our results showed that a possible methodological source of the observed heterogeneity was the year of data collection. Between-study heterogeneity may be influenced by the data collection year because more recent investigations provide information about younger cohorts of older persons who might have benefited from better access to healthcare, while people from older cohorts may not have had such access [13].

### Limitations and strengths of the study

This review included a number of studies conducted in different cities and different countries; thus, caution is required when interpreting the results. The high level of heterogeneity among the studies may be related to the research designs of the primary studies selected by this review, different sample sizes, health statuses, and cultural, demographic, and socioeconomic differences among the countries investigated [24]. Although these countries are in the same region, they are quite disparate in economic and cultural terms. Consequently, these discrepancies might influence the heterogeneity observed between studies. The unbalanced distribution of studies among the three American regions is another consideration factor when interpreting the results. We could not assess frailty distributions by age due to study differences when reporting the age categories. Publication bias might have been present in this review, although we have tried to understand it using the “trim and fill” approach.

One of the strengths of this review is that an extensive search of studies was carried out in databases and in the grey literature with the aim of diminishing the risk of losing studies (selection bias). Moreover, possible causes of heterogeneity were investigated through meta-regression and sensitivity and subgroup analyses to allow for a better understanding of the high variability between studies. In addition, authors from the selected original studies were contacted for gathering additional data for this review.

Frailty is a topic that requires further investigation. Although the population of older people in LAC is growing fast and need attention, there are not enough investigations regarding the subject in the region. Future studies should detail the prevalence of frailty in each Latin American and Caribbean country as well as in the region as a whole to obtain more precise estimates. Moreover, consensus regarding the use of a unified tool for measuring frailty is needed, as it would allow more comprehensive comparisons to be made between primary studies.

This systematic review analyzed the available literature regarding the prevalence of frailty in people living in an under-researched region. It revealed that roughly one in five community-dwelling older persons is frail in LAC. This is a massive estimate in a region of fragile institutions where the population has been aging rapidly, and it is predicted to continue growing. Results from this study may assist policy makers and the healthcare community in further investigating frailty and its aspects, as frailty was demonstrated to be very common among older people in LAC.

## Supporting Information

S1 Fig Funnel Plot of the prevalence of frailty in LAC. (TIF)

S2 Fig Trim and fill graph. (TIF)

S3 Fig Meta-regression of the prevalence of frailty in LAC. (TIF)

S1 Table Search strategies. (DOCX)

S2 Table Quality Assessment. (DOCX)

S3 Table Data extraction form. (DOCX)
