# Supplementary figures and images for: Prevalence of Frailty in Latin America and the Caribbean: A Systematic Review and Meta-Analysis
Source: PLoS One. 2016 Aug 8;11(8):e0160019. doi: 10.1371/journal.pone.0160019 (PMC4976913; doi:10.1371/journal.pone.0160019)

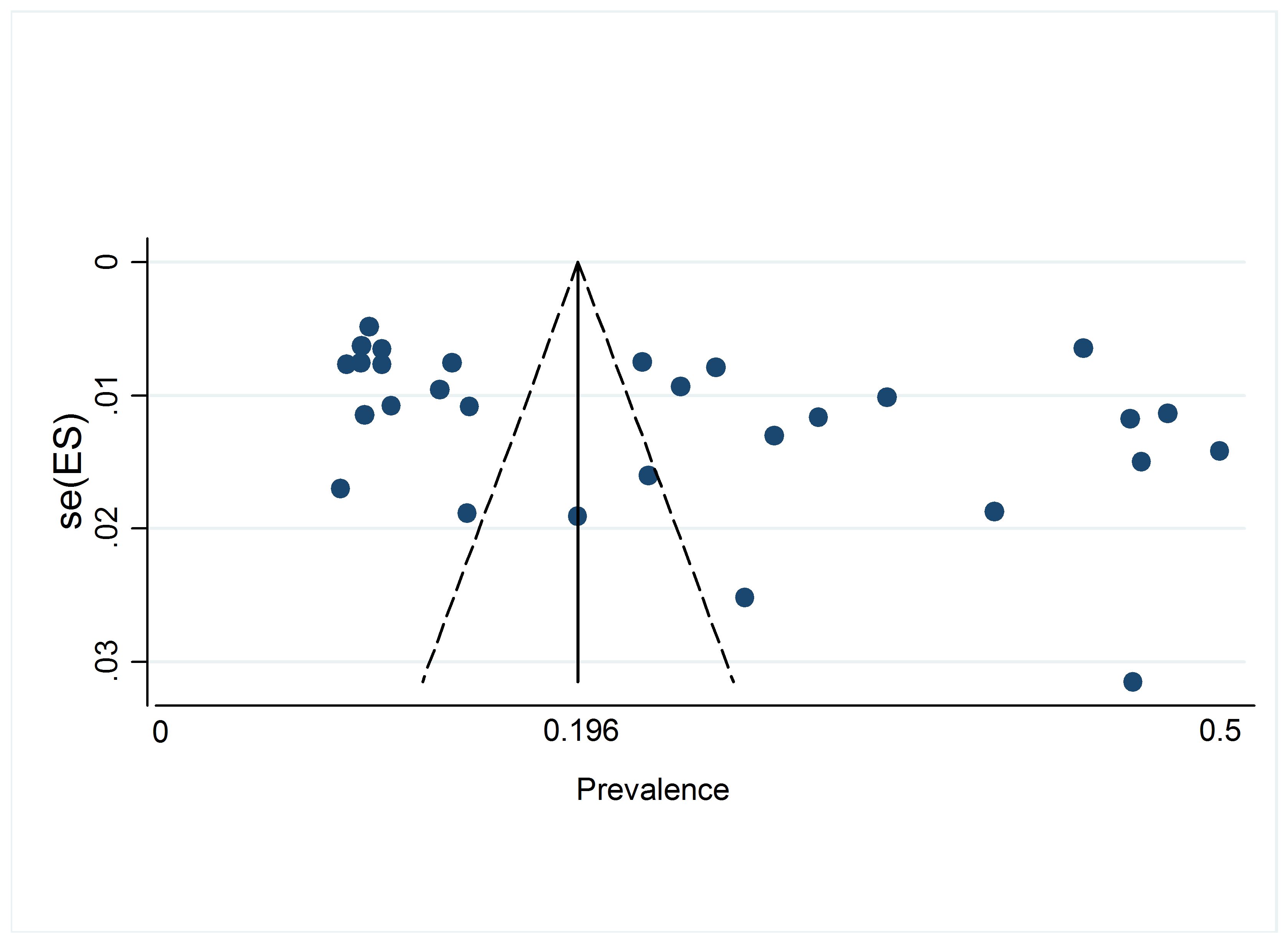

Supplement: S1 Fig — (TIF) [file pone.0160019.s001.tif]

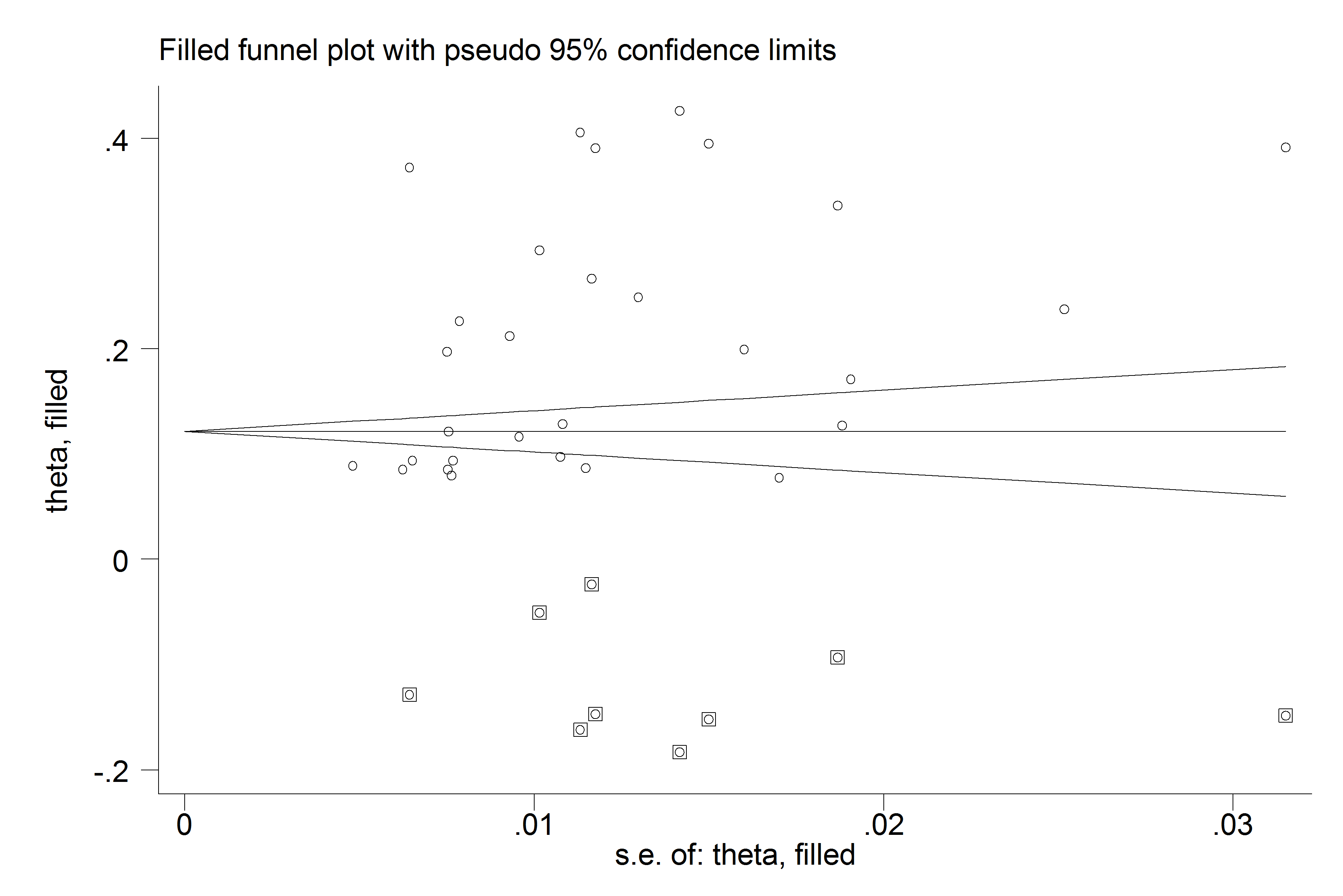

Supplement: S2 Fig — (TIF) [file pone.0160019.s002.tif]

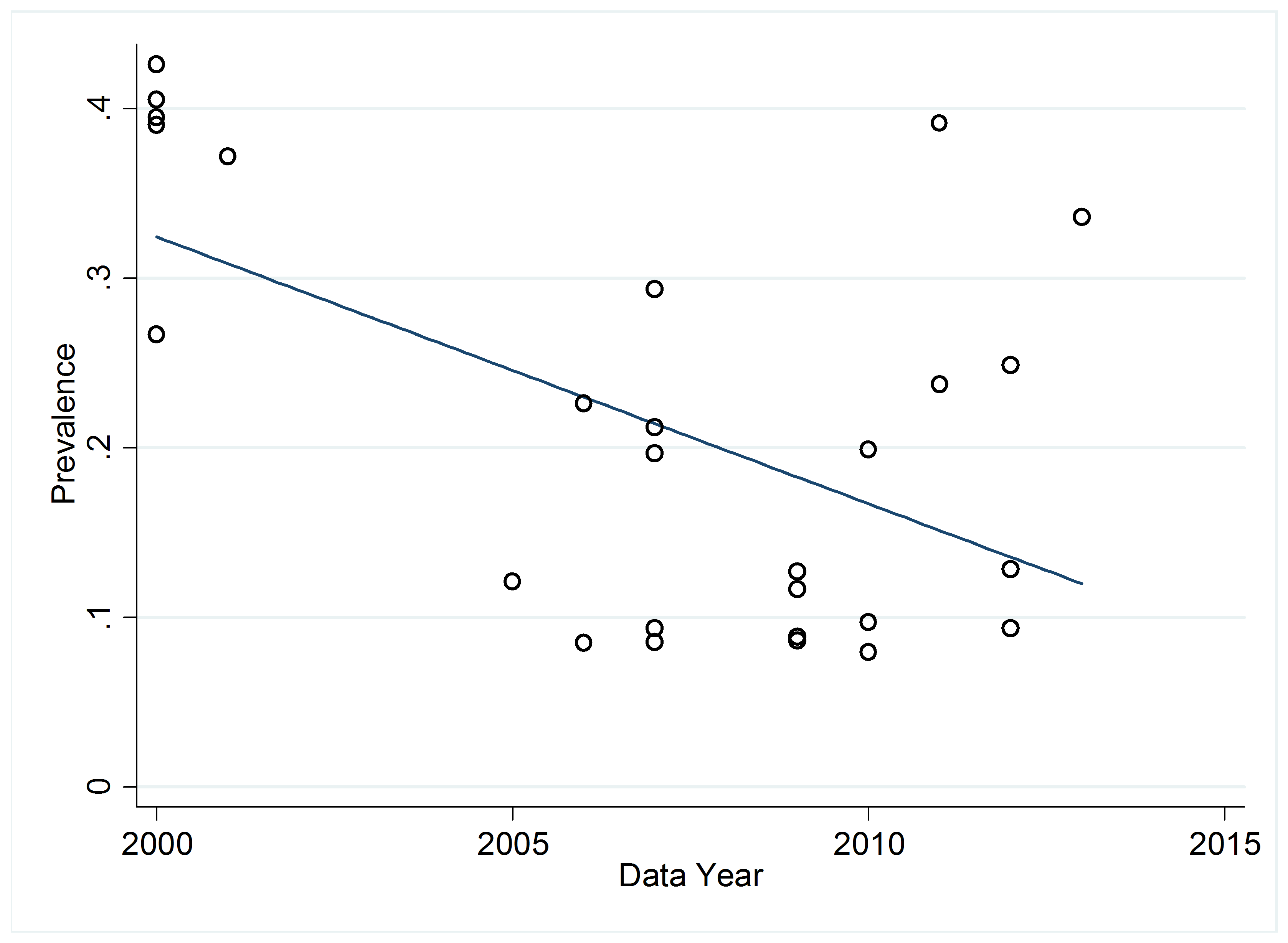

Supplement: S3 Fig — (TIF) [file pone.0160019.s003.tif]
