# Supplementary material for: Prevalence of Frailty in Latin America and the Caribbean: A Systematic Review and Meta-Analysis
Source: PLoS One. 2016 Aug 8;11(8):e0160019. doi: 10.1371/journal.pone.0160019 (PMC4976913; doi:10.1371/journal.pone.0160019)
Supplement: S1 Table — (DOCX) [file pone.0160019.s004.docx]

| **Database** | **Search Strategy** |
| --- | --- |
| **MEDLINE**  **(via PubMed)** | (“Frail Elderly” [Mesh] OR "Frail Elderly” [TIAB] OR “Frailty” OR “Frail Older People”) AND ("Prevalence" OR “Frequency”) |
| **EMBASE** | #2 #1 AND [embase]/lim NOT [medline]/lim  #1 'frail elderly/exp' OR 'frailty' OR 'frail older people' OR 'frail older adults' AND ('prevalence'/exp OR ‘prevalence’ OR ‘frequency/exp OR 'prevalence' OR 'frequency'/  exp OR 'frequency') |
| **LILACS** | Frail Elderly OR Frailty OR Frail Older People [Palavras] and Prevalence OR Frequency [Palavras] |
| **SciELO** | (Frail elderly OR frailty OR frail older people) AND (prevalence OR frequency) |
| **Web of Science** | ((("Frail Elderly” OR “Frailty” OR “Frail Older People”) AND ("Prevalence" OR “Frequency”))) |
| **Scopus** | ( ( frail elderly OR frailty OR frail older people ) AND ( prevalence OR frequency ) ) |
| **CINAHL** | (Frail Elderly OR Frail Elderly OR Frailty OR Frail Older People) AND (Prevalence OR Frequency) |
| **PROQUEST** | (("frail elderly" OR "frailty" OR "frail older people") AND ("prevalence" or "frequency"))  Applied filters:  - Location:  Brazil OR Mexico OR Latin America OR Central America OR Chile OR Puerto Rico OR Sao Paulo Brazil  - Database:  ProQuest Research Library OR ProQuest Research Library: Health & Medicine OR ProQuest Dissertations & Theses Global: Health & Medicine |
| **Academic works** | ("Fragilidade em idosos" OR "fragilidade de idosos" OR "fragilidade") |
| **Google Scholar** | (“Frail Elderly” OR "Frail Elderly” OR “Frailty” OR “Frail Older People”) AND ("Prevalence" OR “Frequency”) AND (“Latin America”) AND (“Caribbean”) |
