# Supplementary material for: Prevalence of Frailty in Latin America and the Caribbean: A Systematic Review and Meta-Analysis
Source: PLoS One. 2016 Aug 8;11(8):e0160019. doi: 10.1371/journal.pone.0160019 (PMC4976913; doi:10.1371/journal.pone.0160019)
Supplement: S2 Table — (DOCX) [file pone.0160019.s005.docx]

| **Study author and year** | **1. Was the sample representative of the target population?** | **2. Were study participants recruited in an appropriate way?** | **3. Was the sample size adequate?** | **4. Were the study subjects and setting described in detail?** | **5. Was the data analysis conducted with sufficient coverage of the identified sample?** | **6. Were valid methods used for the identification of the condition?** | **7. Was the condition measured in a standard, reliable way for all participants?** | **8. Was there appropriate statistical analysis?** | **9. Was the response rate adequate, and if not, was the low response rate managed appropriately?** | **Total number of “yes”** |
| --- | --- | --- | --- | --- | --- | --- | --- | --- | --- | --- |
| Aguilar-Navarro, et al. 2015 [94] | Yes | Yes | Yes | Yes | Yes | Yes | Unclear | Yes | Yes | 8 |
| Alvarado, et al. 2008 [29] | Yes | Yes | Yes | Yes | Yes | Yes | Yes | Yes | Yes | 9 |
| Andrade, et al. 2013 [95] | Yes | Yes | Yes | Yes | Yes | Yes | Yes | Yes | Yes | 9 |
| Corona, et al. 2015 [96] | Yes | Yes | Yes | Yes | Yes | Yes | Yes | Yes | Yes | 9 |
| Curcio, et al. 2014 [97] | No | Unclear | Yes | Yes | Yes | Yes | Yes | Yes | Yes | 7 |
| Fohn, et al. 2013[98] | Yes | Yes | Yes | Yes | Yes | Yes | Yes | Yes | Yes | 9 |
| García-Peña, et al. 2016 [99] | Yes | Yes | Yes | Yes | Yes | Yes | Yes | Yes | Yes | 9 |
| Jotheeswaran, et al. 2015 [30] | Yes | Yes | Yes | Yes | Yes | Yes | Yes | Yes | Yes | 9 |
| Junior, et al. 2014 [100] | Yes | Yes | Yes | Yes | Yes | Yes | Unclear | Yes | Yes | 8 |
| Neri, et al. 2013 [101] | Yes | Yes | Yes | Yes | Yes | Yes | Yes | Yes | Yes | 9 |
| Ocampo-Chaparro, etal. 2013 [102] | Yes | Yes | Yes | Yes | Yes | Yes | Yes | Yes | Yes | 9 |
| Pegarori, et al. 2014 [103] | Yes | Unclear | Yes | Yes | Yes | Yes | Yes | Yes | Yes | 8 |
| Pinedo, et al. 2010 [104] | Yes | Unclear | Unclear | Yes | Yes | Yes | Yes | Yes | Yes | 7 |
| Ramos, et al. 2015 [105] | Yes | Yes | Yes | Yes | Yes | Yes | Yes | Yes | Yes | 9 |
| Ricci, et al. 2014 [106] | Yes | Yes | Yes | Yes | Yes | Yes | Yes | Yes | Yes | 9 |
| Rosero-Bixby, et al. 2009 [107] | Yes | Yes | Yes | Yes | Yes | Yes | Yes | Yes | Yes | 8 |
| Ruiz-Arregui, et al. 2013 [108] | Unclear | No | Yes | Yes | Yes | Yes | Yes | Yes | Yes | 7 |
| Samper-Ternent, et al. 2016 [109] | Yes | Unclear | Yes | Yes | Yes | Yes | Yes | Yes | Yes | 8 |
| Sousa , et al. 2012 [110] | Yes |  | Yes | Yes | Yes | Yes | Yes | Yes | Yes | 9 |
| Tribess, et al. 2012 [111] | Yes | Not given | Yes | Yes | Yes | Yes | Unclear | Yes | Yes | 7 |
| Vieira, et al. 2013 [112] | Yes | Yes | Yes | Yes | Yes | Yes | Not given | Yes | Yes | 8 |
