# Supplementary material for: Prevalence of Frailty in Latin America and the Caribbean: A Systematic Review and Meta-Analysis
Source: PLoS One. 2016 Aug 8;11(8):e0160019. doi: 10.1371/journal.pone.0160019 (PMC4976913; doi:10.1371/journal.pone.0160019)
Supplement: S3 Table — (DOCX) [file pone.0160019.s006.docx]

| **Author, publication year** | **Database** | **Title** | **Country** | **Study group** | **Study design** | **Year of data collection** | **Frailty definition** | **Sample size (n)** | **Mean age** | **Women (%)** | **Frailty Prevalence (%)** | **Confidence Interval (CI)** | **N frail** | **N frail women** | **N frail men** | **N total women** | **N total men** |
| --- | --- | --- | --- | --- | --- | --- | --- | --- | --- | --- | --- | --- | --- | --- | --- | --- | --- |
| Aguilar-Navarro et al., 2015 [94] | Web of Science | Frailty among Mexican community-dwelling elderly: a story told 11 years later. The Mexican Health and Aging Study | Mexico | Mexican Health and Aging Study (MHAS) | Baseline of a longitudinal study | 2001 | Modified version of Frailty Phenotype | 5,644 | 68.7 | 53.6 | 37.2 | NA | 2,100 | 265 | 121 | 921 | 583 |
| Alvarado et al., 2008 [29] | PubMed | Life course social and health conditions linked to frailty in Latin American older men and women. | Barbados | SABE | Population-based | 1999-2000 | Modified version of Frailty Phenotype | 1,446 | NA | 61.0 | 26.7 | NA | 386 | 491 | 271 | 1,262 | 881 |
| Alvarado et al., 2008 [29] | PubMed | Life course social and health conditions linked to frailty in Latin American older men and women. | Brazil | SABE | Population-based | 1999-2000 | Modified version of Frailty Phenotype | 1,879 | NA | 59.0 | 40.6 | NA | 762 | 389 | 131 | 855 | 446 |
| Alvarado et al., 2008 [29] | PubMed | Life course social and health conditions linked to frailty in Latin American older men and women. | Chile | SABE | Population-based | 1999-2000 | Modified version of Frailty Phenotype | 1,22 | NA | 65.7 | 42.6 | NA | 520 | 505 | 169 | 1,197 | 708 |
| Alvarado et al., 2008 [29] | PubMed | Life course social and health conditions linked to frailty in Latin American older men and women. | Cuba | SABE | Population-based | 1999-2000 | Modified version of Frailty Phenotype | 1,726 | NA | 62.8 | 39.0 | NA | 674 | 420 | 128 | 740 | 507 |
| Alvarado et al., 2008 [29] | PubMed | Life course social and health conditions linked to frailty in Latin American older men and women. | Mexico | SABE | Population-based | 1999-2000 | Modified version of Frailty Phenotype | 1,063 | NA | 56.4 | 39.5 | NA | 420 | 78 | 38 | 820 | 554 |
| Andrade et al., 2013 [95] | PubMed | Relationship between oral health and frailty in community-dwelling elderly individuals in Brazil. | Brazil | SABE - São Paulo | Cross-sectional | 2006 | Modified version of Fried Phenotype | 1,374 | NA | 59.7 | 8.5 | NA | 117 | NA | NA | NA | NA |
| Corona et al., 2015 [96] | PubMed | The Relationship between Anemia, Hemoglobin Concentration and Frailty in Brazilian Older Adults. | Brazil | SABE - São Paulo | Cross-sectional | 2010 | Modified version of Fried Phenotype | 1,256 | 70.0 | 60.9 | 8.0 | 6.3-10.2 | 100 | 167 | 61 | 981 | 897 |
| Curcio et al., 2014 [97] | PubMed | Frailty among rural elderly adults. | Colombia | NA | Survey | 2005 | Modified version of Frailty Phenotype | 1,878 | 70.9 | 52.2 | 12.2 | 6.8-17.0 | 228 | NA | NA | NA | NA |
| Fohn et al., 2013 [98] | PubMed | Prevalence of falls among frail elderly adults | Brazil | NA | Cross-sectional | 2010-2011 | Edmonton Frail Scale | 240 | 73.5 | 62.9 | 39.2 | NA | 94 | 157 | 119 | 606 | 502 |
| García-Peña et al., 2016 [99] | PubMed | Frailty prevalence and associated factors in the Mexican health and aging study:  A comparison of the frailty index and the phenotype. | Mexico | Mexican Health and Ageing Study (MHAS) | Cross-sectional | 2012 | Modified version of Fried Phenotype | 1,108 | 69.8 | 54.6 | 24.9 | NA | 276 | NA | NA | NA | NA |
| Jotheeswaran et al., 2015 [30] | Embase | Frailty and the prediction of dependence and mortality in low- and  middle-income countries: A 10/66 population-based cohort study | Cuba | 10/66 Dementia Research Group’s | Population-based | 2003-2007 | Modified version of Frailty Phenotype | 2,813 | 75.2 | 65.0 | 21.0 | NA | 554 | NA | NA | NA | NA |
| Jotheeswaran et al., 2015 [30] | Embase | Frailty and the prediction of dependence and mortality in low- and  middle-income countries: A 10/66 population-based cohort study | Domican Republic | 10/66 Dementia Research Group’s | Population-based | 2003-2007 | Modified version of Frailty Phenotype | 2,011 | 75.4 | 66.3 | 34.6 | NA | 591 | NA | NA | NA | NA |
| Jotheeswaran et al., 2015 [30] | Embase | Frailty and the prediction of dependence and mortality in low- and  middle-income countries: A 10/66 population-based cohort study | Venezuela | 10/66 Dementia Research Group’s | Population-based | 2003-2007 | Modified version of Frailty Phenotype | 1,997 | 72.3 | 63.2 | 11.0 | NA | 187 | NA | NA | NA | NA |
| Jotheeswaran et al., 2015 [30] | Embase | Frailty and the prediction of dependence and mortality in low- and  middle-income countries: A 10/66 population-based cohort study | Mexico | 10/66 Dementia Research Group’s | Population-based | 2003-2007 | Modified version of Frailty Phenotype | 2,003 | 74.2 | Urban population: 66.5 Rural population: 60.9 | Urban population: 10.1 Rural population: 8.5 | NA | 171 | NA | NA | NA | NA |
| Jotheeswaran et al., 2015 [30] | Embase | Frailty and the prediction of dependence and mortality in low- and  middle-income countries: A 10/66 population-based cohort study | Peru | 10/66 Dementia Research Group’s | Population-based | 2003-2007 | Modified version of Frailty Phenotype | 1,933 | 74.5 | Urban population: 64.7 Rural population: 53.2 | Urban population: 25.9 Rural population: 17.2 | NA | 410 | NA | NA | NA | NA |
| Junior et al., 2914 [100] | PubMed | Pre-frailty and frailty of elderly residents in a municipality with a low Human Development Index. | Brazil | Nutritional status, risk behaviors and health conditions of the elderly people of Lafaiete Coutinho-BA. | Cross-sectional | 2011 | Modified version of Frailty Phenotype | 286 | NA | NA | 23.8 | NA | 68 | 224 | 84 | 2,355 | 1123 |
| Neri et al., 2013 [101] | LILACS | Methodology and social, demographic, cognitive, and frailty profiles of community-dwelling elderly from seven Brazilian cities: the FIBRA Study | Brazil | FIBRA NETWORK | Cross-sectional | 2008-2009 | Fried Phenotype (CHS) | 3,478 | 72.9 | 67.7 | 9.0 | NA | 308 | 27 | 13 | 202 | 112 |
| Ocampo-Chaparro et al., 2013 [102] | LILACS | Prevalence of poor self-rated health and associated risk factors among older adults in Cali, Colombia | Colombia | NA | Population-based | 2009 | Modified version of Frailty Phenotype | 314 | NA | NA | 12.7 | NA | 40 | 91 | 32 | 617 | 341 |
| Pegarori et al., 2014 [103] | Citation reference | Fatores associados à síndrome de fragilidade em idosos residentes em área urbana | Brazil | FIBRA NETWORK | Cross-sectional | 2012 | Modified version of Frailty Phenotype | 958 | 73.8 | 64.4 | 12.8 | 10.87-15.11 | 123 | 16 | 3 | 147 | 99 |
| Pinedo et al., 2010 [104] | PubMed | Gait speed as an indicator of fragility in community-dwelling elders in Lima, Peru. | Peru | NA | Cross-sectional | NA | Modified version of Frailty Phenotype | 246 | 69.9 | 59.8 | 7.7 | NA | 19 | NA | NA |  |  |
| Ramos et al., 2015 [105] | Embase | Prevalence of depressive symptoms and associated factors among elderly in  northern Minas Gerais: A population-based study | Brazil | NA | Population-based | 2013 | Edmonton Frail Scale | 639 | 70.6 | 64.0 | 33.6 | NA | 215 | 49 | 25 | 489 | 272 |
| Ricci et al., 2014 [106] | Citation reference | Frailty and cardiovascular risk in community-dwelling elderly: a population based study | Brazil | FIBRA NETWORK | Population-based | 2009-2010 | Fried Phenotype (CHS) | 761 | 71.9 | 64.3 | 9.7 | NA | 74 | 486 | 153 | 1,418 | 1290 |
| Rosero-Bixby et al., 2009 [107] | PubMed | Surprising SES Gradients in mortality, health, and biomarkers in a Latin American population of adults. | Costa Rica | CRELES | Baseline of a longitudinal study | 2004-2006 | Five physical tests: grip strength, pulmonary peak flow, standing up from a chair, picking an object up from the floor, and standing and walking 3m | 2,827 | NA | 52.4 | 23.6 | 21.1-26.3 | 639 | 85 | 46 | 509 | 418 |
| Ruiz-Arregui et al., 2013 [108] | PubMed | The Coyoacán Cohort Study: Design, Methodology, and Participants' Characteristics of a Mexican Study on Nutritional and Psychosocial Markers of Frailty. | Mexico | Coyoacán Cohort Study | Baseline of a longitudinal study | 2008-2009 | Modified version of Frailty Phenotype | 927 | 79.5 | 54.9 | 14.1 | 11.9-16.5 | 131 | 88 | 47 | 880 | 562 |
| Samper-Ternent et al., 2016 [109] | PubMed | Frailty and sarcopenia in Bogota´: results from the SABE Bogota´ Study | Colombia | SABE (Bogotá Study) | Cross-sectional | 2012 | Modified version of Frailty Phenotype | 1,442 | 70.7 | 61.0 | 9.4 | NA | 135 | 44 | 23 | 240 | 151 |
| Sousa et al., 2012 [110] | PubMed | Frailty syndrome and associated factors in community-dwelling elderly in Northeast Brazil. | Brazil | FIBRA Network | Cross-sectional | NA | Fried Phenotype (CHS) | 391 | 74.0 | 61.4 | 17.1 | NA | 67 | 81 | 43 | 404 | 218 |
| Tribess et al., 2012 [111] | PubMed | Physical activity as a predictor of absence of frailty in the elderly. | Brazil | Population Study of Physical Activity and Aging (*Estudo Populacional de Atividade Física e Envelhecimento*) | Cross-sectional | 2010 | Modified version of Frailty Phenotype | 622 | 71.0 | 65.0 | 19.9 | NA | 124 | NA | NA | NA | NA |
| Vieira et al., 2013 [112] | PubMed | Prevalence of frailty and associated factors in community-dwelling elderly in Belo Horizonte, Minas Gerais State, Brazil: data from the FIBRA study | Brazil | FIBRA NETWORK | Population-based | 2008-2009 | Fried Phenotype (CHS) | 601 | 74.3 | 66.2 | 8.7 | NA | 52 | NA | NA | NA | NA |
